# 

**Published:** 1910-02

**Authors:** 


### BOOKS RECEIVED.

---

Heart Disease, Blood Pressure and the Nauheim-Scott Treatment. By Louis Faugeres Bishop, A.M., M.D., Clinical Professor of Heart and Circulatory Diseases, Fordham University, School of Medicine, New York. Third edition. 12 mo., pp. 284. Illustrated. New York: E. B. Treat & Co. 1909. (Cloth, $3.00.)

Vital Economy or How to Conserve Your Strength. By John H. Clarke, M.D. 12 mo., pp. 119. New York: A. Wessels. 1909. (Cloth, 50 cents.)

Textbook of Medical and Pharmaceutical Chemistry. By Elias H. Bartley, B.S., M.D., Ph.G., Professor of Chemistry, Toxicology and Pediatrics in Long Island College Hospital. Seventh edition. 12 mo., pp. 734. Illustrated. Philadelphia: P. Blakiston's Son & Co. 1909. (Cloth, $3.00 net.)

Examination of the Urine: A Manual for Students and Practitioners. By G. A. DeSantos Saxe, M.D., Instructor in Genitourinary Surgery, New York Post-Graduate Medical School and Hospital. Second edition, enlarged and reset. 12 mo., of 448 pages, illustrated. Philadelphia and London: W. B. Saunders Company. 1909. (Cloth, $1.75 net.)

International Clinics. A Quarterly of Illustrated Clinical Lectures and especially prepared articles on Treatment, Medicine, Surgery, Neurology, Pediatrics, Obstetrics, Gynecology, Orthopedics, Pathology, Dermatology, Ophthalmology, Otology, Rhinology, Laryngology, Hygiene and other topics of interest to students and practitioners. Edited by W. T. Longcope, M.D. Volume IV. Nineteenth series. Philadelphia and London. J. B. Lippincott Co. 1909. (Cloth, $2.00.)

The Practical Medicine Series. Ten volumes. Under the general ediorial charge of Gustavus P. Head, M.D., Professor of Laryngology and Rhinology in the Chicago Post-Graduate Medical School. Vol. X. Nervous and Mental Diseases. Edited by Hugh T. Patrick, M.D., and Charles L. Mix, M.D. Series 1909. Chicago: The Year Book Publishers. (Price, $1.25; entire series, $10.00.)

